# Supplementary material for: A comparison of viral strategies and model systems to target norepinephrine neurons in the locus coeruleus reveals high variability in transgene expression patterns
Source: PLoS Biol. 2025 Jul 7;23(7):e3003228. doi: 10.1371/journal.pbio.3003228 (PMC12233902; doi:10.1371/journal.pbio.3003228)
Supplement: S1 Table — Bold font indicates mouse lines used in this study. Gray font indicates mouse lines which are not readily available from any vendor. (DOCX) [file pbio.3003228.s003.docx]

**S1 Table | Cre driver lines to target the NE system in mice.** Bold font indicates mouse lines used in this study. Grey font indicates mouse lines which are not readily available from any vendor.

| *strain name* | *trivial name* | *gene* | *strategy* | *provider* | *strain ID* | *reference* |
| --- | --- | --- | --- | --- | --- | --- |
| B6.Cg-Dbh^tm3.2(cre)Pjen^/J | ***Dbh^cre^*** | Dopamine-β-Hydroxylase | knock-in/ knock-out | *Jackson Laboratory* | 033951 | Tillage et al.^19^ |
| Tg(Dbh-cre)KH210Gsat/Mmucd | *Dbh^cre^* | Dopamine-β-Hydroxylase | BAC | *Mutant Mouse Resource & Research Centers* | 036734-UCD | Gong et al.^18^ |
| Tg(Dbh-cre)KH212Gsat/Mmucd | *Dbh^cre^* | Dopamine-β-Hydroxylase | BAC | *Mutant Mouse Resource & Research Centers* | 032081-UCD | Gong et al.^18^ |
| B6.Cg-7630403G23 RikTg*^(Th-cre)1Tmd^*/J | ***Th^cre^*** | Tyrosine Hydroxylase | random insertion | *Jackson Laboratory* | 008601 | Savitt et al.^22^ |
| B6.FVB(Cg)Tg(Th-cre)Fl172Gsat/Mmucd | *Th^cre^* | Tyrosine Hydroxylase | BAC | *Mutant Mouse Resource & Research Centers* | 031029-UCD | Gong et al.^18^ |
| B6.129X1-Th^tm1(cre)Te^/Kieg | *Th^cre^* | Tyrosine Hydroxylase | Targeted mutation | *European Mouse Mutant Archive* | EM: 00254 | Lindeberg et al.^23^ |
| Tg(Slc6a2-cre)#Stl | ***Net^cre^*** | Norepi-nephrine Transporter | BAC | – | – | Wagatsuma et al.^21^ |
