# Supplementary material for: A comparison of viral strategies and model systems to target norepinephrine neurons in the locus coeruleus reveals high variability in transgene expression patterns
Source: PLoS Biol. 2025 Jul 7;23(7):e3003228. doi: 10.1371/journal.pbio.3003228 (PMC12233902; doi:10.1371/journal.pbio.3003228)
Supplement: S2 Table — The table indicates results of a two-way ANOVA analyzing effects of genotype (GT), sex, or interactions thereof (×) in the mouse lines included in this study on different aspects of behavior in the in the open field test (OF), elevated plus maze (EPM), Y-maze (YM), and Morris water maze (MWM). All groups included eight male and eight female mice of each genotype. p-values below 0.05 are indicated in bold font. Note that multiple comparisons were not corrected in this table. (DOCX) [file pbio.3003228.s004.docx]

**S2 Table | Statistical analysis of behavioral screening.** The table indicates results of a two-way ANOVA analyzing effects of genotype (GT), sex, or interactions thereof (×) in the mouse lines included in this study on different aspects of behavior in the in the open field test (OF), elevated plus maze (EPM), Y-maze (YM), and Morris water maze (MWM). All groups included 8 male and 8 female mice of each genotype. *p*-values below 0.05 are indicated in bold font. Note that multiple comparisons were not corrected in this table.

| paradigm | | *Dbh^cre^* | | | *Net^cre^* | | | *Th^cre^* | | |
| --- | --- | --- | --- | --- | --- | --- | --- | --- | --- | --- |
| test | **readout** | **GT** | **Sex** | **×** | **GT** | **Sex** | **×** | **GT** | **Sex** | **×** |
| OF | *t* in center | *F* = 0.45  *p* = 0.51 | *F* = 0.39  *p* = 0.22 | *F* = 1.60  *p* = 0.22 | *F* = 0.54  *p* = 0.47 | *F* = 3.93  *p* = 0.06 | *F* = 0.01  *p* = 0.94 | *F* = 0.01  *p* = 0.91 | *F* = 4.22  *p* = 0.05 | *F* = 4.37  *p* = 0.05 |
|  | center crossings | *F* = 0.01  *p* = 0.91 | *F* = 0.41  *p* = 0.54 | *F* = 1.87  *p* = 0.28 | *F* = 0.02  *p* = 0.89 | *F* = 0.14  *p* = 0.71 | *F* = 0.05  *p* = 0.82 | *F* = 0.01  *p* = 0.91 | *F* = 1.34  *p* = 0.26 | *F* = 0.33  *p* = 0.57 |
|  | latency to center | *F* = 1.33  *p* = 0.26 | *F* = 1.00  *p* = 0.33 | *F* = 0.11  *p* = 0.74 | *F* = 1.27  *p* = 0.27 | *F* = 0.29  *p* = 0.60 | *F* = 1.00  *p* = 0.33 | *F* = 1.10  *p* = 0.34 | *F* = 2.96  *p* = 0.10 | *F* = 2.89  *p* = 0.10 |
|  | distance to wall | *F* = 0.61  *p* = 0.44 | *F* = 6.91  ***p* = 0.01** | *F* = 3.26  *p* = 0.08 | *F* = 0.73  *p* = 0.4 | *F* = 2.31  *p* = 0.54 | *F* = 0.39  *p* = 0.14 | *F* = 3.34  *p* = 0.08 | *F* = 2.09  *p* = 0.16 | *F* = 6.41  ***p* = 0.02** |
|  | *t* in border | *F* = 1.52  *p* = 0.23 | *F* = 11.7  ***p* < 0.01** | *F* = 1.44  *p* = 0.24 | *F* = 2.81  *p* = 0.11 | *F* = 1.82  *p* = 0.19 | *F* = 0.07  *p* = 0.79 | *F* = 1.74  *p* = 0.20 | *F* = 0.37  *p* = 0.55 | *F* = 3.72  *p* = 0.06 |
|  | travel distance | *F* = 0.03  *p* = 0.87 | *F* = 0.28  *p* = 0.60 | *F* = 0.03  *p* = 0.87 | *F* = 0.54  *p* = 0.47 | *F* = 3.93  *p* = 0.06 | *F <* 0.01  *p* = 0.94 | *F* = 0.01  *p* = 0.94 | *F* = 16.4  ***p* < 0.01** | *F <* 0.01  *p* = 0.97 |
| EPM | *t* in open arm | *F* = 1.96  *p* = 0.17 | *F* = 1.95  *p* = 0.17 | *F* = 0.01  *p* = 0.91 | *F <* 0.01  *p* = 0.93 | *F* = 0.76  *p* = 0.39 | *F* = 2.34  *p* = 0.14 | *F* = 0.32  *p* = 0.57 | *F* = 0.07  *p* = 0.79 | *F* = 0.12  *p* = 0.73 |
|  | open arm entries | *F* = 1.91  *p* = 0.22 | *F* = 0.01  *p* = 0.91 | *F* = 0.17  *p* = 0.68 | *F* = 0.75  *p* = 0.60 | *F* = 0.07  *p* = 0.80 | *F* = 0.21  *p* = 0.65 | *F* = 0.14  *p* = 0.71 | *F* = 0.03  *p* = 0.87 | *F* = 0.33  *p* = 0.57 |
|  | open arm edges | *F* < 0.01  *p* = 0.94 | *F* = 5.52  ***p* = 0.03** | *F* = 0.30  *p* = 0.59 | *F* < 0.01  *p* = 0.95 | *F* = 3.57  *p* = 0.07 | *F* = 0.16  *p* = 0.69 | *F* < 0.01  *p* = 0.95 | *F* = 3.57  *p* = 0.07 | *F* = 0.16  *p* = 0.69 |
|  | *t* to leave center | *F* = 0.01  *p* = 0.91 | *F* = 5.02  *p* = 0.03 | *F* < 0.01  *p* = 0.98 | *F* = 2.59  *p* = 0.12 | *F* = 2.12  *p* = 0.16 | *F* < 0.01  *p* = 0.94 | *F* = 4.19  *p* = 0.05 | *F* = 0.61  *p* = 0.44 | *F* = 0.12  *p* = 0.73 |
|  | Rearing events | *F* = 2.13  *p* = 0.16 | *F* = 1.45  *p* = 0.16 | *F* = 2.13  *p* = 0.16 | *F* = 0.81  *p* = 0.38 | *F* = 3.20  *p* = 0.08 | *F <* 0.01  *p* = 0.93 | *F* = 0.91  *p* = 0.35 | *F* = 2.43  *p* = 0.13 | *F* = 0.12  *p* = 0.73 |
|  | *t* to rearing | *F* = 2.11  *p* = 0.16 | *F* = 1.41  *p* = 0.24 | *F* = 0.04  *p* = 0.85 | *F* = 2.28  *p* = 0.15 | *F* = 2.77  *p* = 0.11 | *F* = 0.95  *p* = 0.34 | *F* = 0.26  *p* = 0.61 | *F* = 0.26  *p* = 0.61 | *F* = 4.43  ***p* = 0.04** |
|  | *t* grooming | *F* = 1.01  *p* = 0.32 | *F* = 0.87  *p* = 0.36 | *F* = 0.05  *p* = 0.82 | *F* = 1.23  *p* = 0.26 | *F* = 0.82  *p* = 0.37 | *F* = 1.98  *p* = 0.17 | *F* = 0.25  *p* = 0.62 | *F* = 0.23  *p* = 0.63 | *F* = 1.42  *p* = 0.24 |
| YM | alter-nations | *F* = 1.30  *p* = 0.26 | *F* = 1.62  *p* = 0.21 | *F* = 0.24  *p* = 0.62 | *F* = 2.48  *p* = 0.13 | *F* = 2.32  *p* = 0.14 | *F* = 0.02  *p* = 0.89 | *F* = 0.23  *p* = 0.64 | *F* = 2.95  *p* = 0.09 | *F* = 0.31  *p* = 0.58 |
|  | *t*/ transitions | *F* = 0.04  *p* = 0.84 | *F* = 0.76  *p* = 0.39 | *F* = 2.55  *p* = 0.12 | *F* = 3.04  *p* = 0.09 | *F* = 2.95  *p* = 0.09 | *F* = 0.31  *p* = 0.56 | *F* = 3.04  *p* = 0.09 | *F* = 0.20  *p* = 0.66 | *F* = 11.2  ***p* < 0.01** |
| MWM  day 2 | *t* in platform | *F* = 0.40  *p* = 0.54 | *F* = 0.16  *p* = 0.69 | *F* = 0.66  *p* = 0.42 | *F* = 0.70  *p* = 0.41 | *F* = 0.07  *p* = 0.79 | *F* = 1.3  *p* = 0.26 | *F* = 0.03  *p* = 0.87 | *F* = 0.93  *p* = 0.34 | *F* < 0.01  *p* = 0.93 |
|  | *t* in quadrant | *F* = 0.48  *p* = 0.49 | *F* < 0.01  *p* = 0.99 | *F* = 0.07  *p* = 0.79 | *F* = 0.23  *p* = 0.64 | *F* = 3.01  *p* = 0.09 | *F* = 0.31  *p* = 0.58 | *F* < 0.01  *p* = 0.95 | *F* = 0.32  *p* = 0.57 | *F* < 0.01  *p* = 0.94 |
|  | *t* to platform | *F* = 0.90  *p* = 0.35 | *F* = 0.06  *p* = 0.81 | *F* = 0.08  *p* = 0.78 | *F* = 0.03  *p* = 0.86 | *F* = 0.32  *p* = 0.58 | *F* = 0.60  *p* = 0.44 | *F* =0.07  *p* = 0.79 | *F* = 4.25  *p* = 0.05 | *F* = 1.53  *p* = 0.23 |
|  | distance to platform | *F* = 0.23  *p* = 0.63 | *F* = 1.63  *p* = 0.21 | *F* = 2.40  *p* = 0.13 | *F* = 0.26  *p* = 0.62 | *F* = 2.96  *p* = 0.10 | *F* < 0.01  *p* = 0.95 | *F* = 0.05  *p* = 0.82 | *F* = 1.30  *p* = 0.26 | *F* = 0.27  *p* = 0.61 |
|  | travel distance | *F* = 0.04  *p* = 0.53 | *F* = 0.29  *p* = 0.60 | *F* = 0.58  *p* = 0.45 | *F* = 0.88  *p* = 0.36 | *F* = 2.48  *p* = 0.13 | *F* = 0.02  *p* = 0.88 | *F* = 0.04  *p* = 0.84 | *F* < 0.01  *p* = 0.99 | *F* = 0.46  *p* = 0.51 |
|  | distance to wall | *F* = 1.57  *p* = 0.24 | *F* = 1.47  *p* = 0.24 | *F* = 1.00  *p* = 0.32 | *F* = 0.03  *p* = 0.87 | *F* < 0.01  *p* = 0.99 | *F* = 0.92  *p* = 0.35 | *F* = 0.86  *p* = 0.36 | *F* = 1.42  *p* = 0.24 | *F* = 0.23  *p* = 0.63 |
| MWM  day 3 | *t* in platform | *F* = 1.14  *p* = 0.29 | *F* = 0.80  *p* = 0.38 | *F* < 0.01  *p* = 0.94 | *F* = 0.56  *p* = 0.46 | *F* = 0.43  *p* = 0.52 | *F* = 0.30  *p* = 0.59 | *F* = 0.34  *p* = 0.56 | *F* = 0.30  *p* = 0.59 | *F* = 0.33  *p* = 0.57 |
|  | *t* in quadrant | *F* = 0.04  *p* = 0.85 | *F* = 1.50  *p* = 0.23 | *F* = 0.41  *p* = 0.52 | *F* = 0.68  *p* = 0.42 | *F* = 0.45  *p* = 0.51 | *F* = 0.25  *p* = 0.62 | *F* = 0.59  *p* = 0.45 | *F* = 0.25  *p* = 0.62 | *F* = 0.16  *p* = 0.69 |
|  | *t* to platform | *F* = 0.07  *p* = 0.80 | *F* = 0.29  *p* = 0.60 | *F* = 0.11  *p* = 0.75 | *F* = 0.14  *p* = 0.71 | *F* = 1.59  *p* = 0.22 | *F* = 0.40  *p* = 0.53 | *F* < 0.01  *p* = 0.97 | *F* = 0.24  *p* = 0.63 | *F* = 0.40  *p* = 0.53 |
|  | distance to platform | *F* = 0.11  *p* = 0.75 | *F* = 0.49  *p* = 0.49 | *F* = 0.01  *p* = 0.91 | *F* = 0.88  *p* = 0.36 | *F* = 0.09  *p* = 0.76 | *F* = 2.84  *p* = 0.10 | *F* = 0.07  *p* = 0.80 | *F* = 4.0  *p* = 0.06 | *F* = 0.04  *p* = 0.83 |
|  | travel distance | *F* = 0.06  *p* = 0.82 | *F* = 0.28  *p* = 0.60 | *F* = 0.55  *p* = 0.47 | *F* = 1.24  *p* = 0.27 | *F* = 0.79  *p* = 0.38 | *F* = 0.05  *p* = 0.82 | *F* = 0.13  *p* = 0.73 | *F* = 0.05  *p* = 0.82 | *F* < 0.01  *p* = 0.93 |
|  | distance to wall | *F* = 2.94  *p* = 0.10 | *F* = 0.17  *p* = 0.69 | *F* = 0.89  *p* = 0.35 | *F* = 0.07  *p* = 0.79 | *F* = 2.28  *p* = 0.14 | *F* = 0.11  *p* = 0.75 | *F* < 0.01  *p* = 0.93 | *F* = 2.87  *p* = 0.10 | *F* = 0.48  *p* = 0.50 |
