# Supplementary figures and images for: A comparison of viral strategies and model systems to target norepinephrine neurons in the locus coeruleus reveals high variability in transgene expression patterns
Source: PLoS Biol. 2025 Jul 7;23(7):e3003228. doi: 10.1371/journal.pbio.3003228 (PMC12233902; doi:10.1371/journal.pbio.3003228)

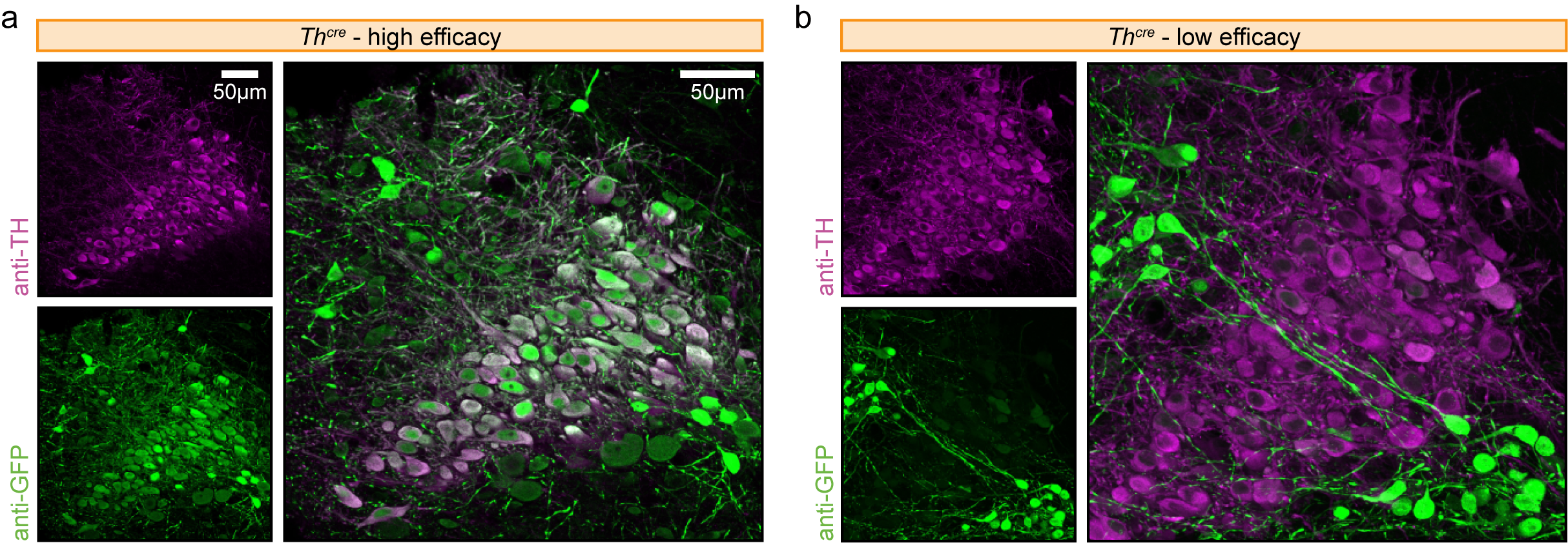

Supplement: S1 Fig — TH-expression (magenta) and eGFP expression (green) in exemplary Thcre mice with high (a) and low efficacy (b). Scale bar: 50 µm. (TIF) [file pbio.3003228.s005.tif]

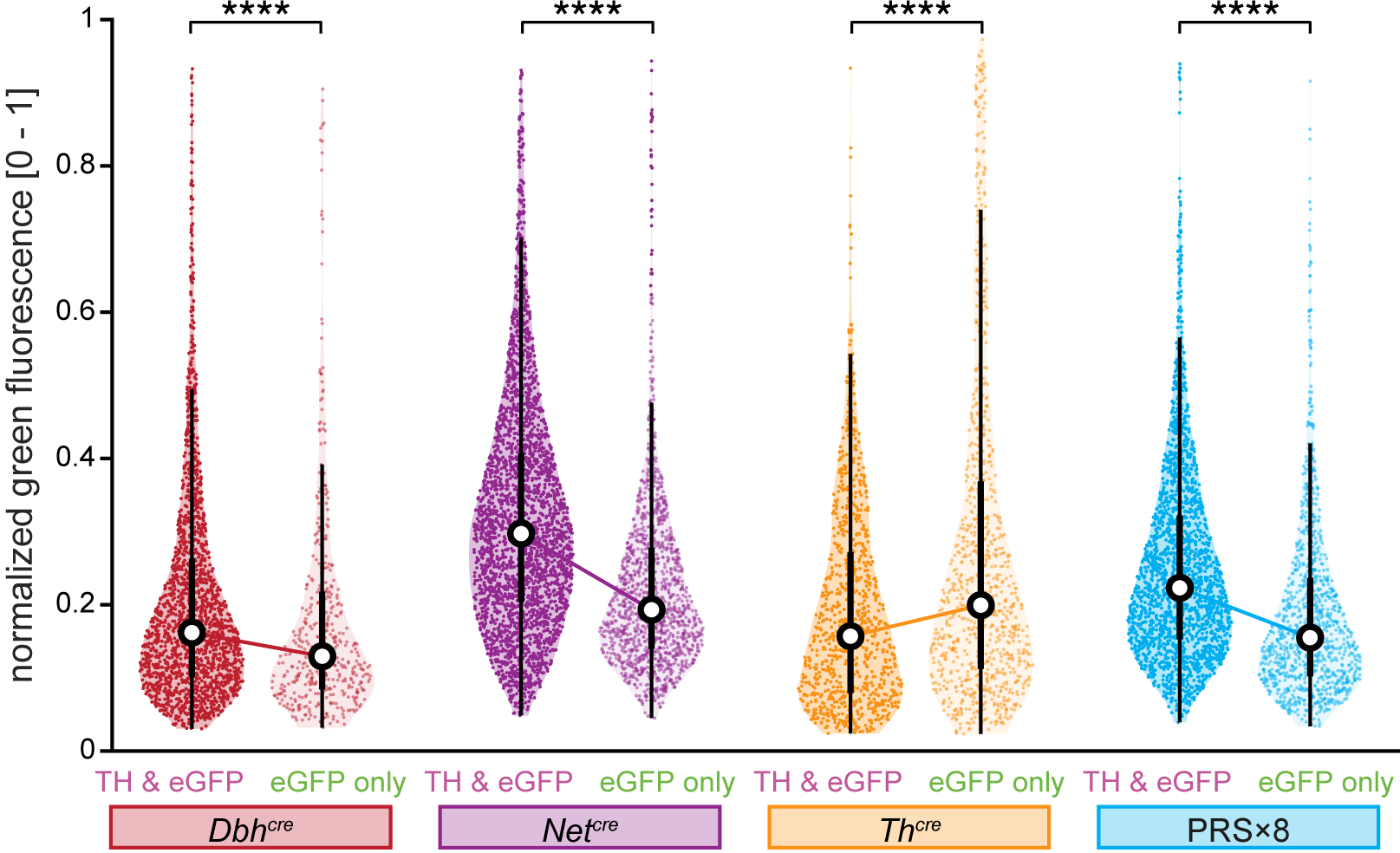

Supplement: S2 Fig — Normalized fluorescence in the green channel of neurons co-expressing TH and eGFP (i.e., true positives; left) and neurons expressing eGFP only (i.e., false positives; right) in Dbhcre (red), Netcre (magenta), Thcre (orange) and PRS×8 mice (blue). While normalized fluorescence of false positive neurons was lower as compared to true positive neurons in Dbhcre, Netcre and PRS×8 mice, normalized fluorescence of false positive neurons exceeded true positive neurons in Thcre mice. ****: p < 0.0001 (Bonferroni-corrected Wilcoxon rank sum test). Numerical data underlying this figure can be found in S1 Data. (TIF) [file pbio.3003228.s006.tif]

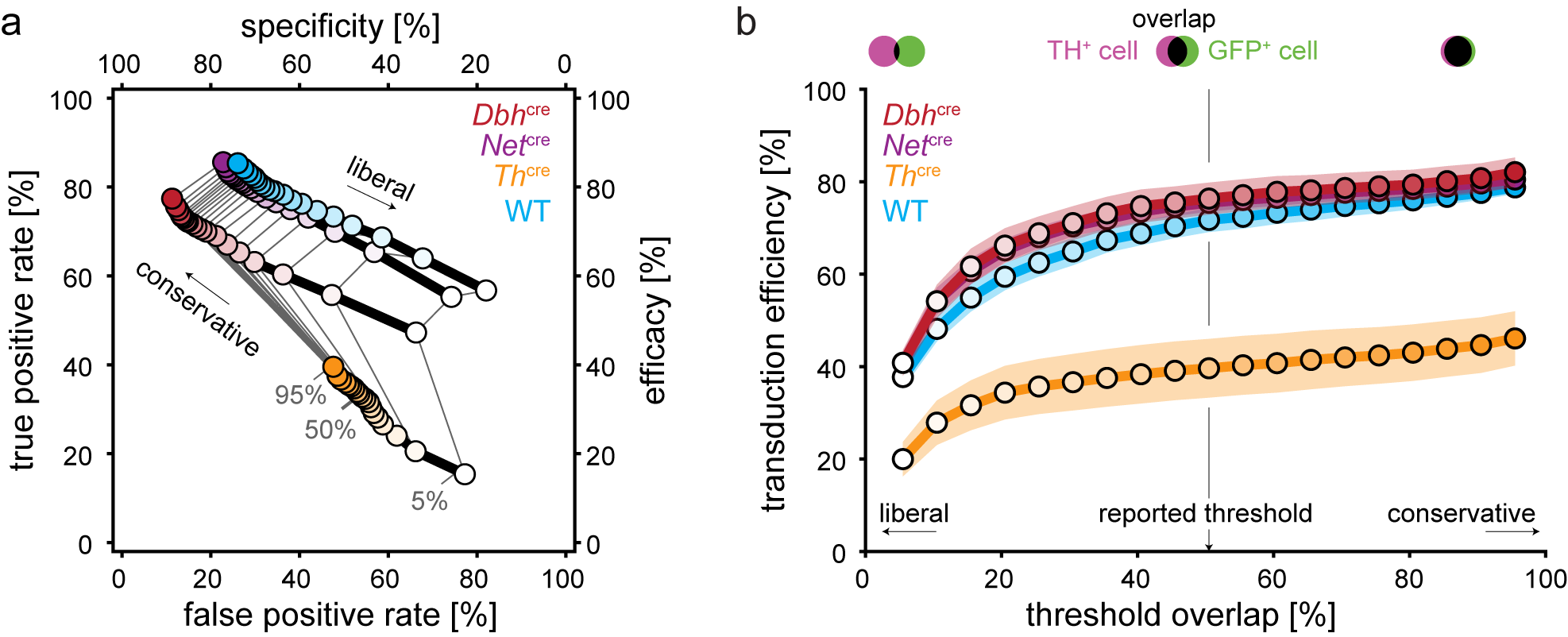

Supplement: S3 Fig — (a) Efficacy and specificity from the four different model systems as a function of the threshold overlap parameter (light to dark colors; ranging from 5% to 95%, in steps of 5%) which defines the minimal overlap between GFP+ and TH+ channels for a TH+ cell to be also labeled as GFP+. (b) Transduction efficiency (summarizing sensitivity and specificity in a single metric) from the four different model systems quantitatively varied as a function of the threshold overlap parameter. Importantly, conclusions were robust to this parameter choice as shown by the non-crossing of the curves. In the main text, we report values for a neutral threshold overlap parameter of 50%. Shading areas denote the standard error of the mean (n = 7). (TIF) [file pbio.3003228.s007.tif]

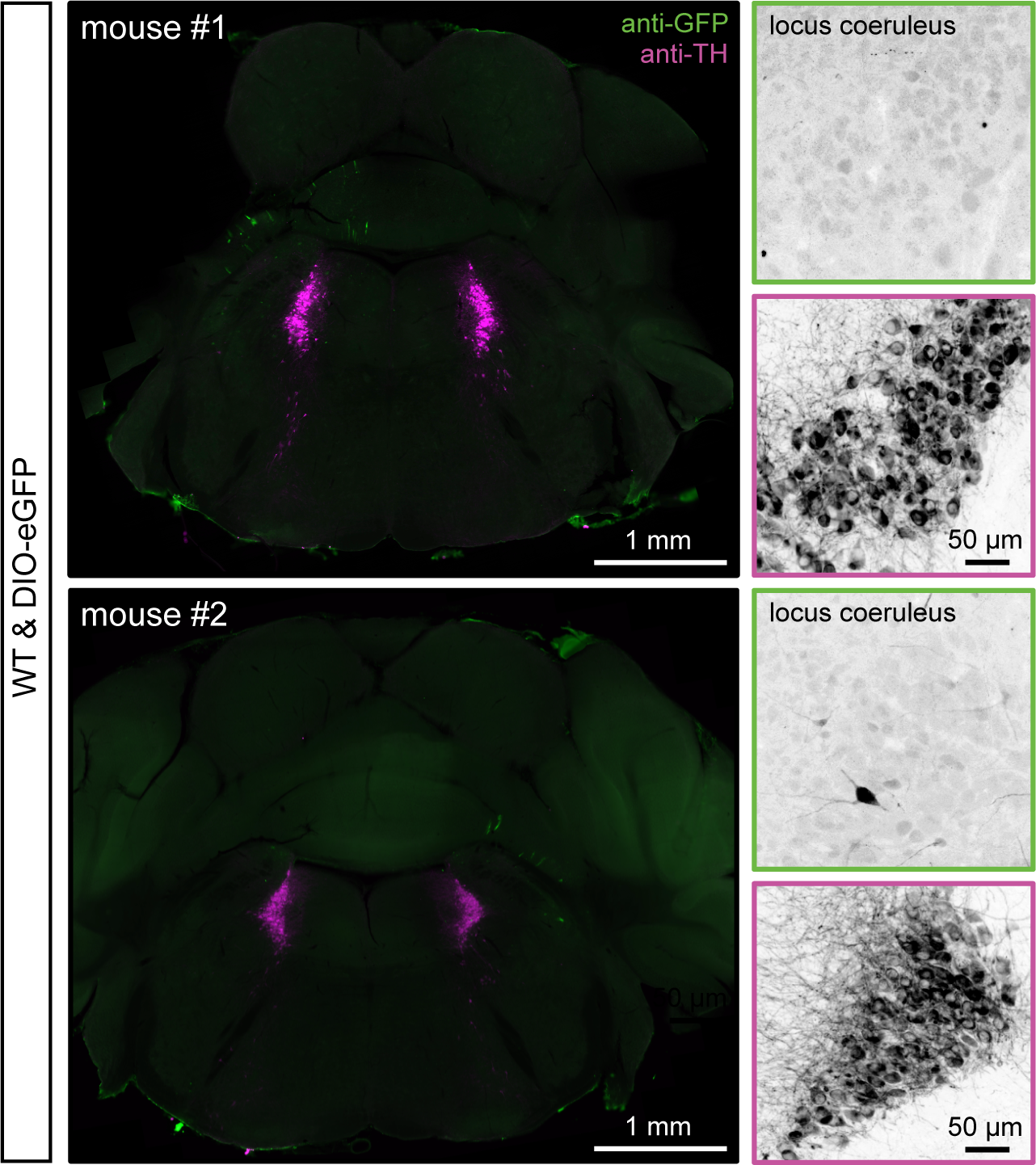

Supplement: S4 Fig — Immunohistochemical staining against GFP (green) and TH (magenta) in two wild-type mice injected with rAAV2/9-DIO-eGFP. eGFP expression was only observed in very few cells both within and outside of the LC. (TIF) [file pbio.3003228.s008.tif]

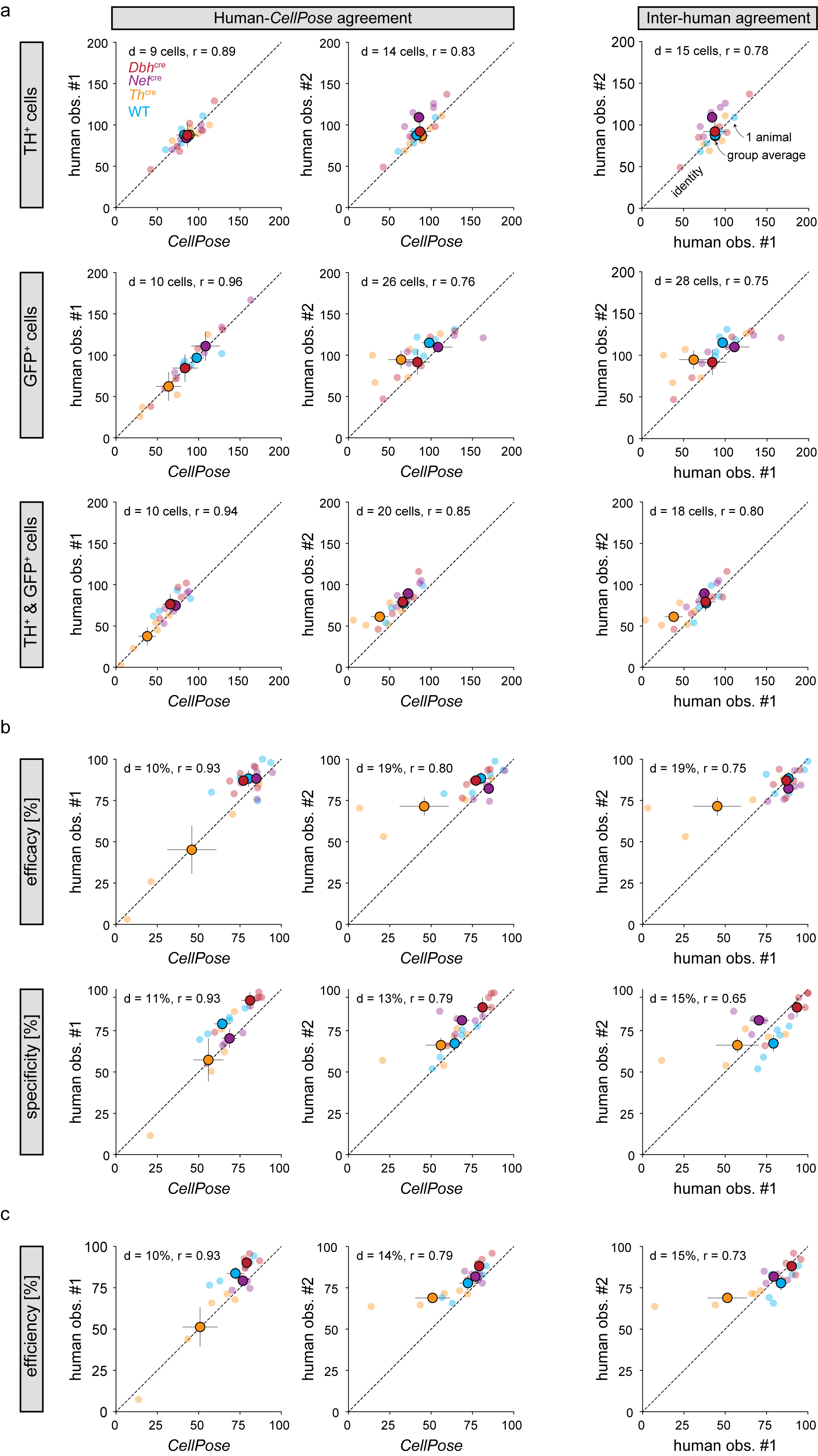

Supplement: S5 Fig — (a) Correlation of cell counts; (b) Efficacy and specificity; (c) transduction efficiency between cells identified by CellPose vs. labeled by each of the two human observers (AD and CW; first 2 columns); and between the two human observers (third column). A total of five slices of five different mice were used for each of the four model systems. Dashed lines indicate identity. In each case, two measures of agreement are reported: Pearson’s correlation coefficient, denoted as r; and the distance between observers, denoted as d = √∑I (xi − yi)2 where x and y correspond to observers’ reports (a) or derived quantities (b, c), and i to the brain slice. Error bars denote the standard error of the mean (with n = 5). (TIF) [file pbio.3003228.s009.tif]

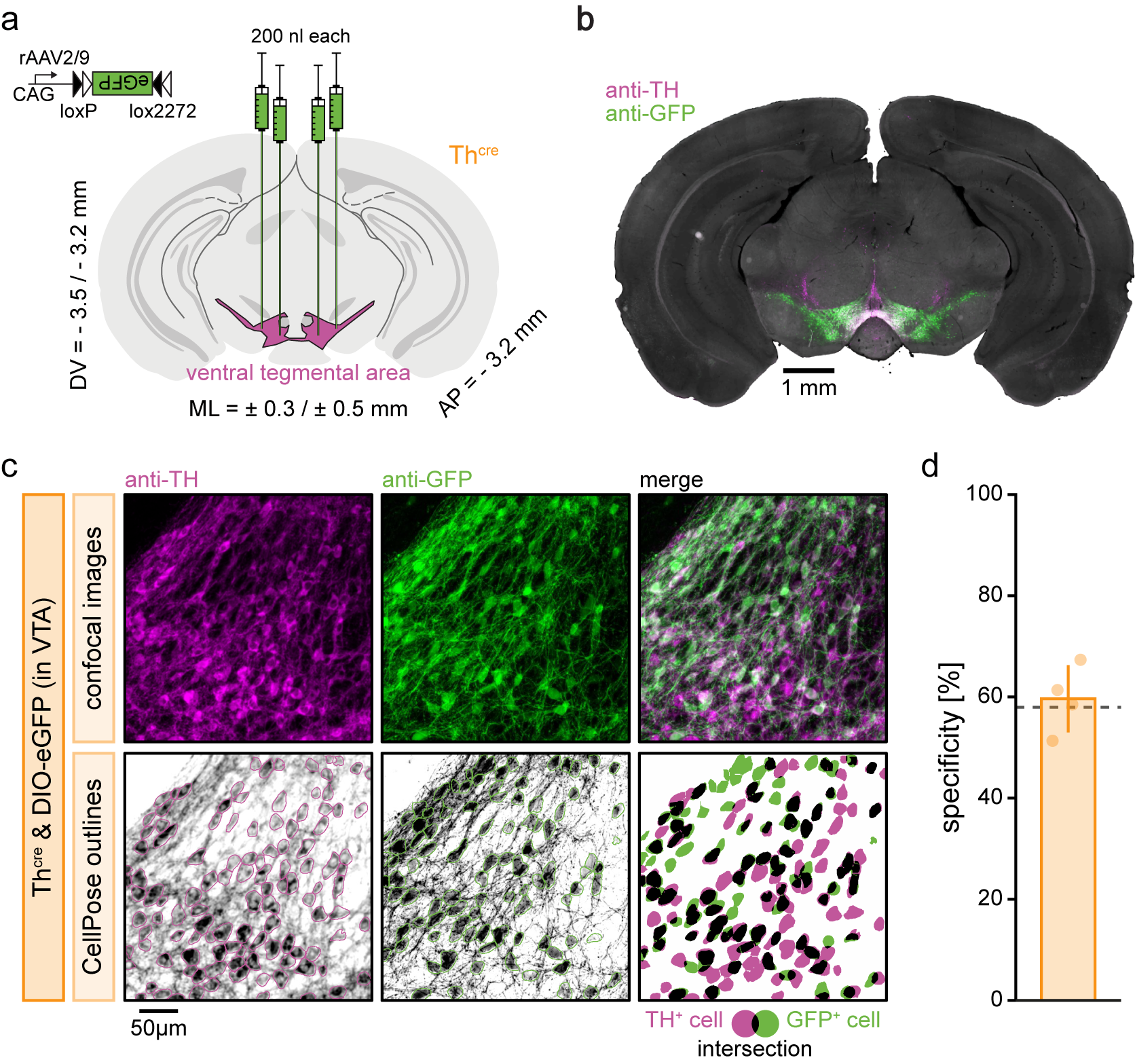

Supplement: S6 Fig — (a) Injection scheme: CAG-DIO-eGFP was bilaterally injected into the VTA of Thcre mice. (b) Example of a coronal brain slice stained against TH (magenta) to visualize dopaminergic neurons of the VTA and eGFP (green) to enhance the native fluorescence of eGFP expression. (c) Exemplary confocal images of VTA-DA neurons in Thcre mice (top), along with corresponding cell masks as segmented by CellPose (bottom). Overlaid cell masks of both channels are shown at the bottom right, where each TH+ cell was labeled as GFP+ (successful transduction, black) when cell masks overlapped 50% or more the size of the TH+ cell, or GFP− otherwise (missed transduction, magenta). GFP+ and TH− (erroneous transduction, green) was counted as unspecific transduction. (d) The average specificity of GFP expression in the VTA of Thcre mice amounted to 61.7 ± 6.8% (derived from a total of 2,469 TH+ and 1,627 eGFP+ neurons obtained from 25 images of n = 4 mice). These findings are in line with the specificity of 59 ± 1% (dashed line) reported by Lammel and colleagues [39] (based on visual inspection). Numerical data underlying panel (d) can be found in S1 Data. (TIF) [file pbio.3003228.s010.tif]

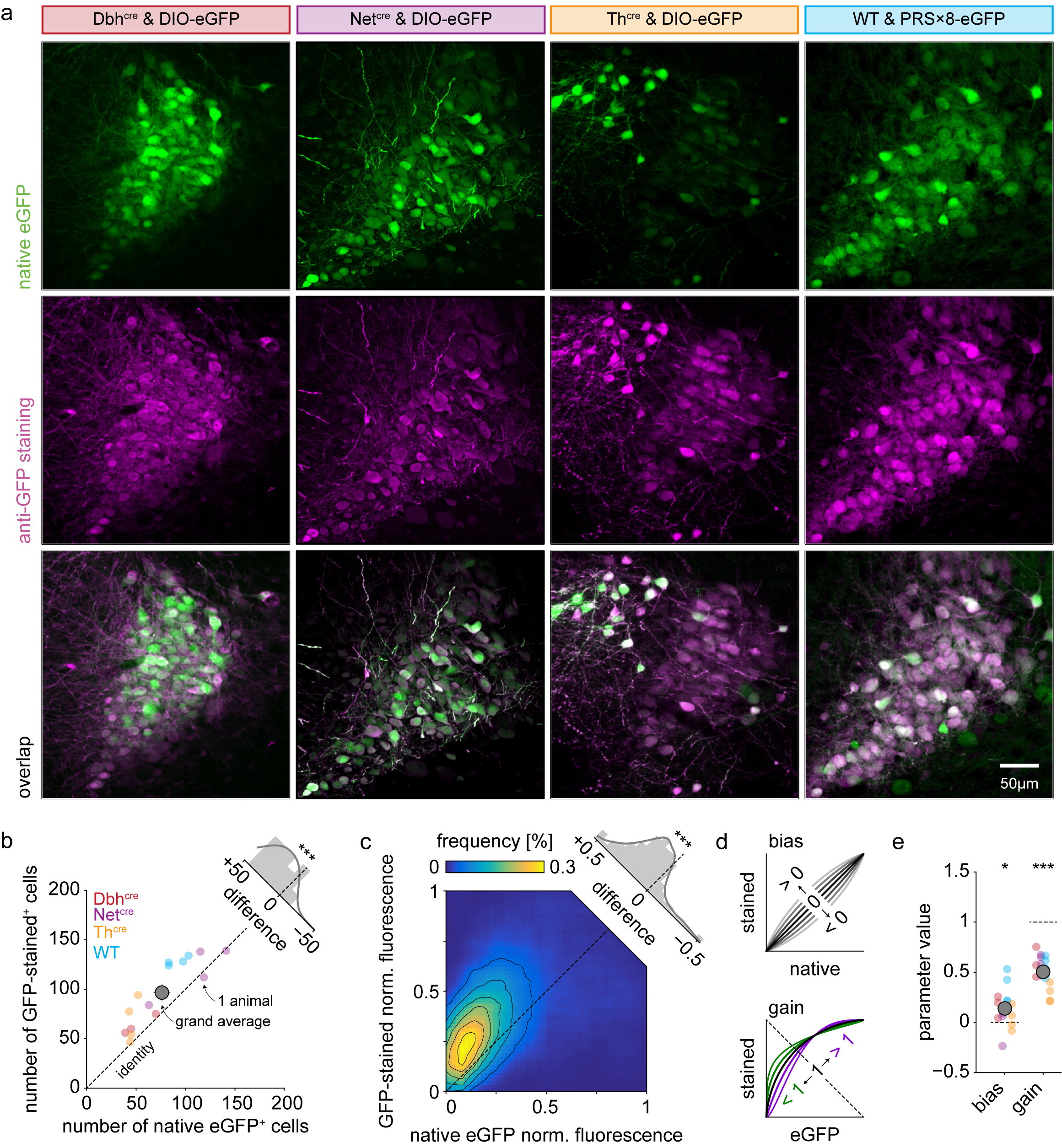

Supplement: S7 Fig — (a) Example images of brain slices showing native eGFP fluorescence (green, top) and anti-GFP immunofluorescence with a secondary antibody in the red spectrum (magenta, center) in Dbhcre, Netcre, Thcre, and PRS×8 mice (from left to right). (b) Number of neurons detected by CellPose in the native eGFP (green, x-axis) and GFP-stained (red, y-axis) channels across animals (n = 3/4/4/4 Dbhcre/Netcre/Thcre/PRS×8 mice, respectively). 21.5 ± 15.3% more cells were detected in the GFP-stained channel as compared to the native eGFP channel (absolute difference: 20.5 ± 16.5 cells; t test against 0: t14 = 4.8, p = 2.8 × 10^−4^). (c) Distribution of normalized fluorescence in anti-GFP stained channel as a function of the normalized fluorescence in the native eGFP channel across cells. Fluorescence was averaged within each cell mask (detected in the GFP-stained channel), then binned into 50 × 50 equally spaced bins, and further smoothed by a 2D 10 × 10 boxcar function. The averaged normalized brightness of immuno-stained cells per animal (0.40 ± 0.06) exceeded the averaged normalized brightness of the native eGFP-expressing cells (0.26 ± 0.06) by 55 ± 35% (absolute difference: 0.13 ± 0.076; t test against 0: t14 = 6.66, p = 1.08 × 10^−5^). (d) To quantify the difference in fluorescence between the anti-GFP immunostaining and native eGFP fluorescence in more detail, we fitted a non-linear function describing the fluorescence of GFP-stained cells as a function of native eGFP fluorescence (after within-channel normalization, see panel c). The function is of the form f′R = ɸ(bias + gain × ɸ−1(fG)), where fR and fG denotes the fluorescence in the red (GFP-stained) and green (native eGFP) channels, respectively, and ɸ corresponds to the normal cumulative distribution function. The ‘bias’ parameter quantifies the overall change in fluorescence induced by the immunostaining (top panel), while the ‘gain’ parameter accounts for a possible asymmetry in the fluorescence increase depending on the [file pbio.3003228.s011.tif]

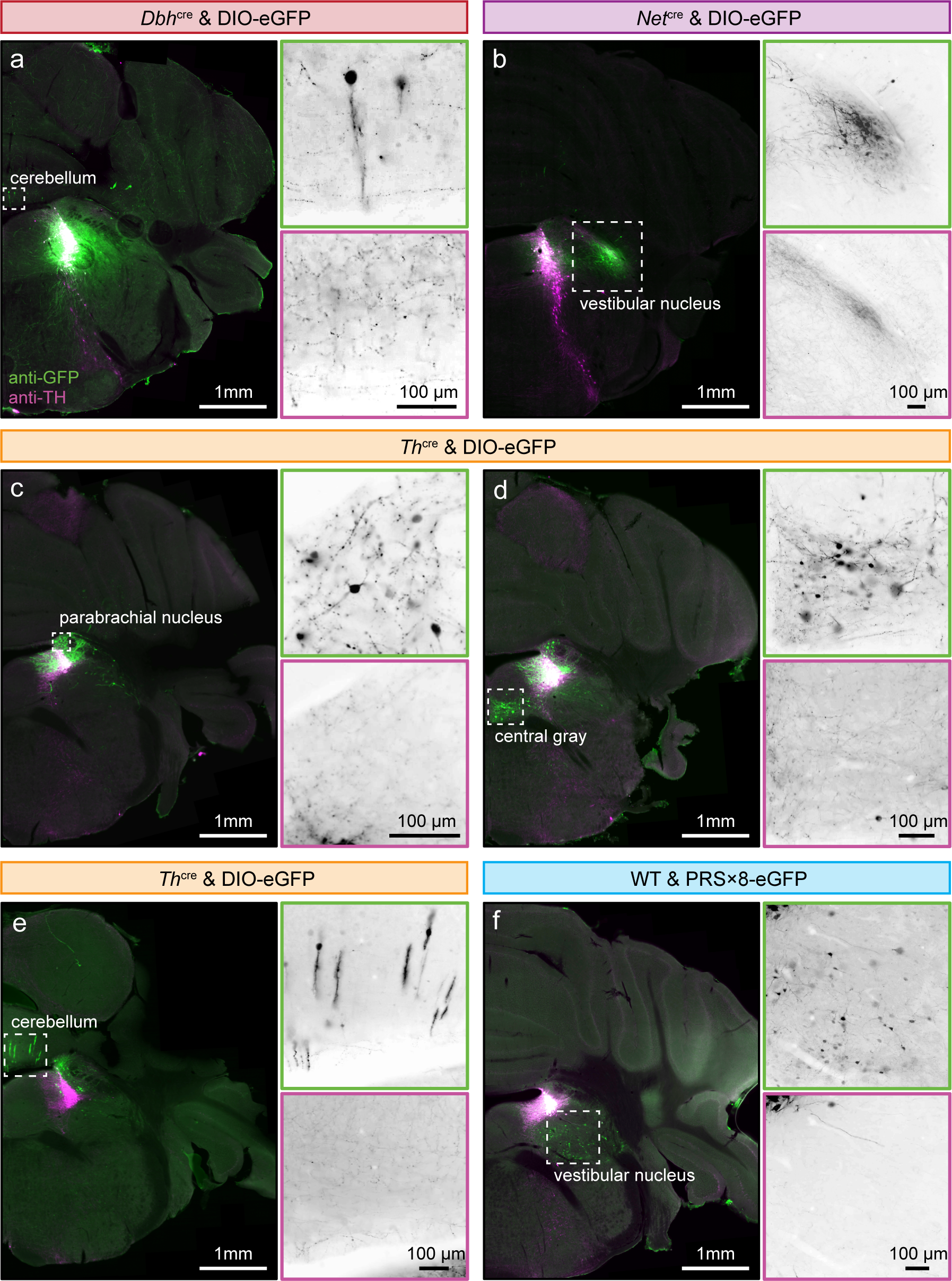

Supplement: S8 Fig — Example brain slices showing ectopic expression upon Dbhcre (a), Netcre (b), Thcre (c–e), and PRS×8 (f) mediated transgene expression. Immuno-straining against TH and GFP is displayed in magenta and green respectively. Insets show inverted gray scale images for a better contrast. (TIF) [file pbio.3003228.s012.tif]

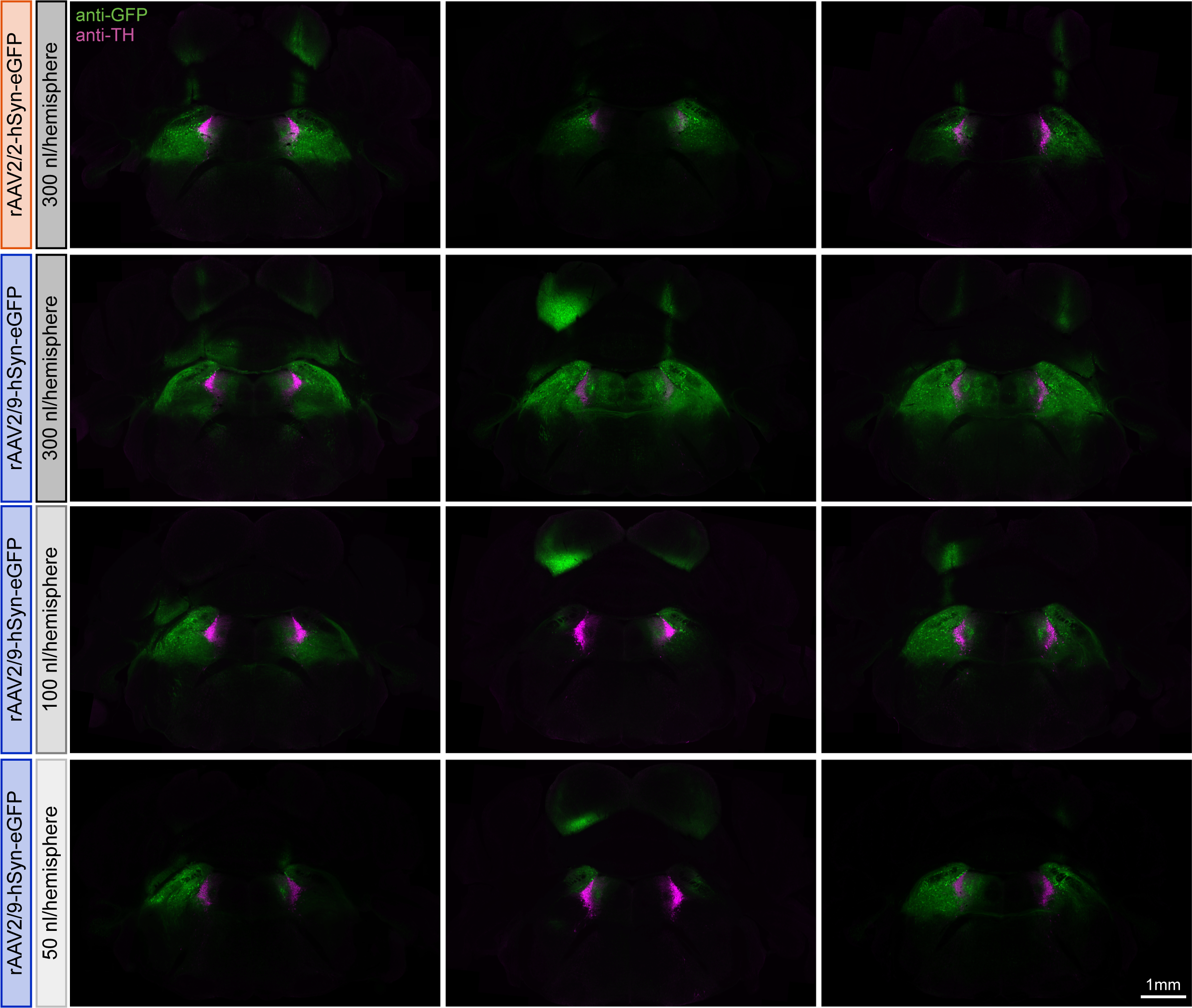

Supplement: S9 Fig — Coronal brain slices of three wild-type mice that were bilaterally injected with 300 nl of rAAV2/2-hSyn-eGFP (top row), 300 nl of rAAV2/9-hSyn-eGFP (second row from top), 100 nl of rAAV2/9-hSyn-eGFP (third row from top), or 50 nl of rAAV2/9-hSyn-eGFP (bottom row). All slices were stained against tyrosine hydroxylase to visualize LC-NE neurons (magenta) and against eGFP to enhance the native fluorescence of transgene expression (green). (TIF) [file pbio.3003228.s013.tif]

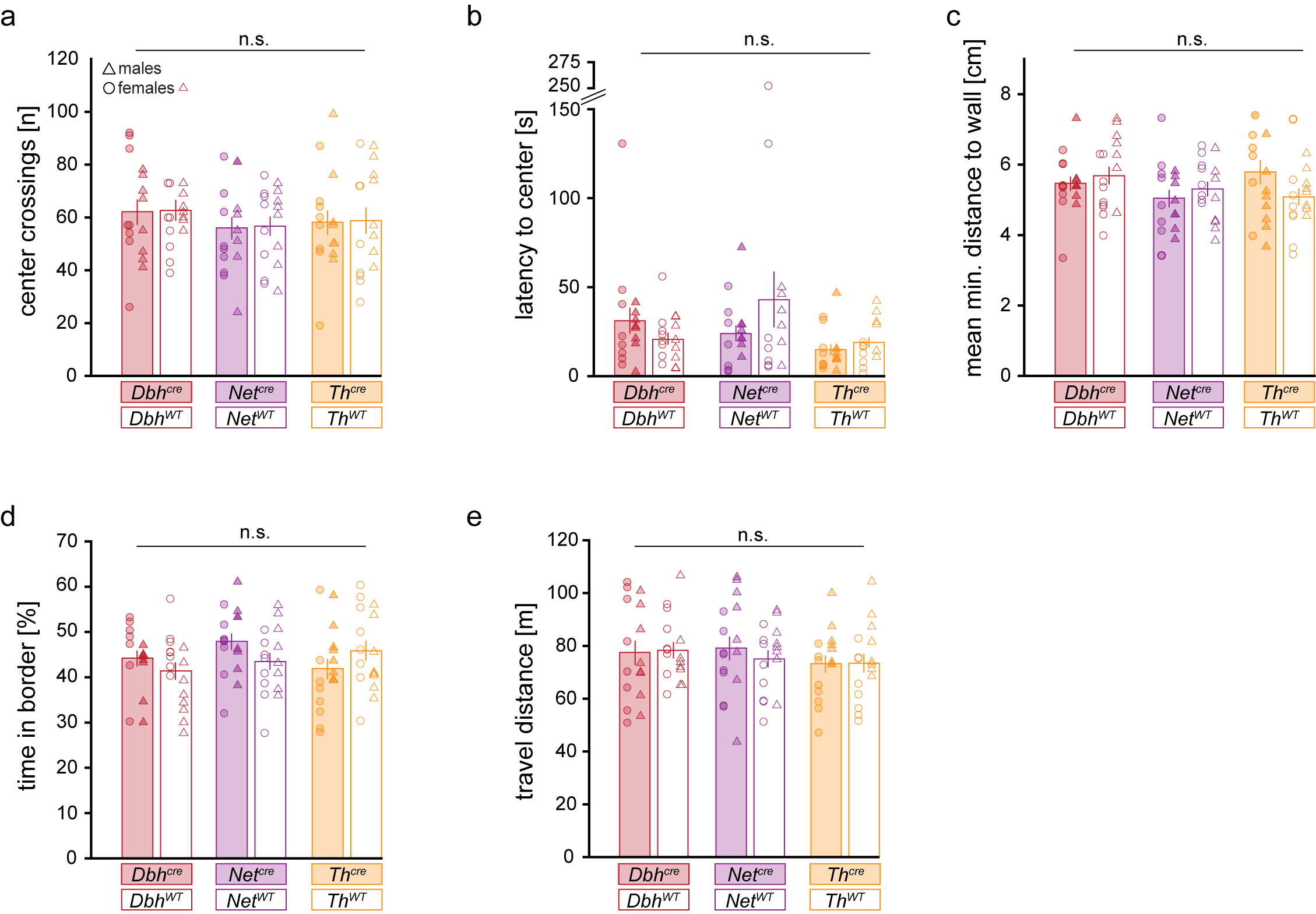

Supplement: S10 Fig — No effect of genotype could be detected on anxiety-like behavior as approximated by (a) the number of center crossings, (b) the latency to the first center crossing, (c) the mean minimum distance to the wall, (d) the percentage of time spent in the border of the arena, nor to general locomotion behavior as approximated by (e) the total distance traveled by the mouse. All data is depicted as mean ± standard error of the mean. n.s. = not significant. n = 8 females (circles) and 8 males (triangles). Numerical data underlying this figure can be found in S1 Data. (TIF) [file pbio.3003228.s014.tif]

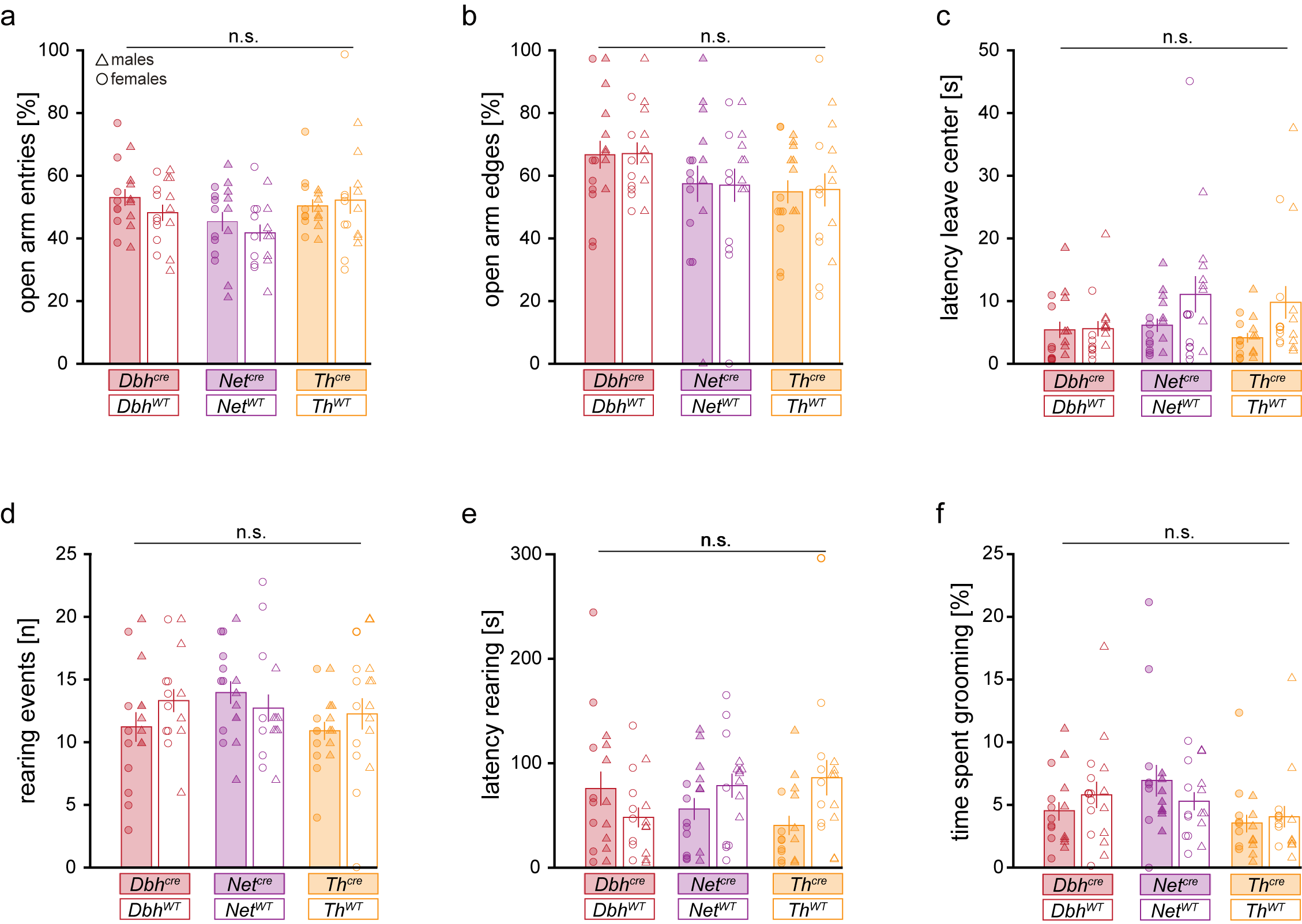

Supplement: S11 Fig — No effect of genotype could be detected on anxiety-like behavior as approximated by (a) the number of open arm entries, (b) the percentage of open arm entries on which the animal went to the distant edge of the arm, (c) the latency to leave the central platform, (d) the number of rearing events, (e) the latency to the first rearing event, or (f) the percentage of time spent grooming. All data is depicted as mean ± standard error of the mean. n.s. = not significant. n = 8 females (circles) and 8 males (triangles). Numerical data underlying this figure can be found in S1 Data. (TIF) [file pbio.3003228.s015.tif]

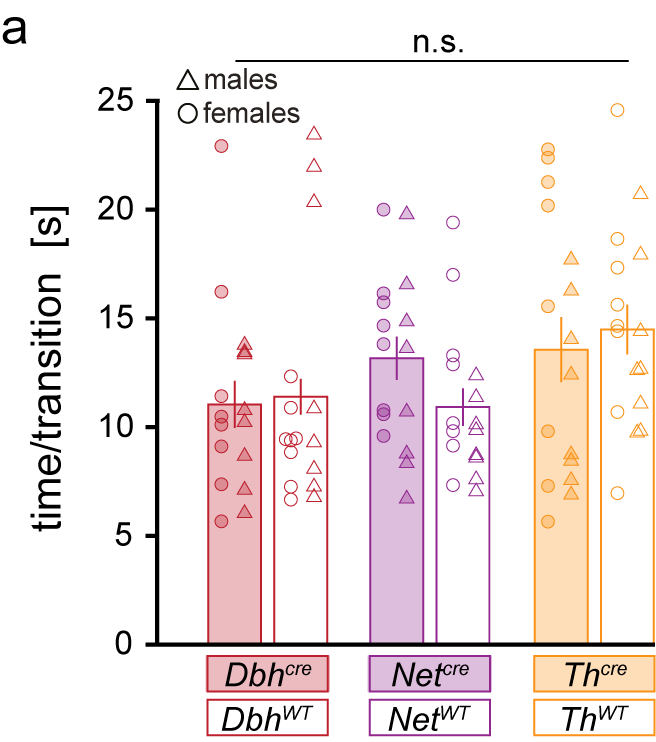

Supplement: S12 Fig — No effect of genotype could be detected on locomotion or explorative behavior as approximated by (a) the average time per transition between Y-maze arms. All data is depicted as mean ± standard error of the mean. n.s. = not significant. n = 8 females (circles) and 8 males (triangles). Numerical data underlying this figure can be found in S1 Data. (TIF) [file pbio.3003228.s016.tif]

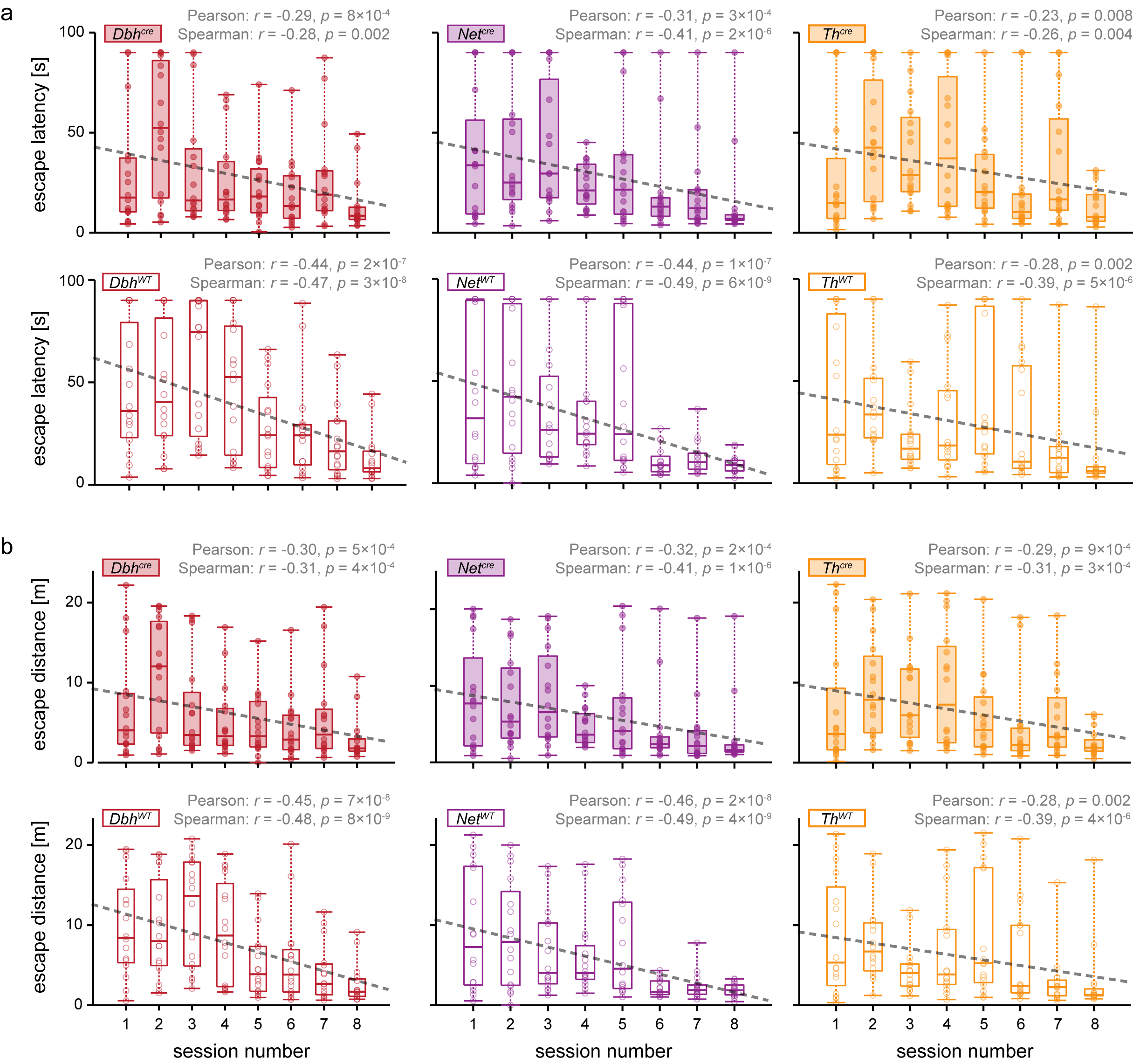

Supplement: S13 Fig — In each group (n = 16 mice/group), both the latency to reach the platform (a) and the distance until the platform was reached (b) decreased as a function of training session number (four sessions were performed on each day for two subsequent days), as indicated by both Pearson’s and Spearman’s correlation, indicating successful spatial learning in the water maze. Boxplots depict 0/25/50/75/100 percentiles, respectively. Dashed lines indicate the linear fit according to the method of least squares. Numerical data underlying this figure can be found in S1 Data. (TIF) [file pbio.3003228.s017.tif]

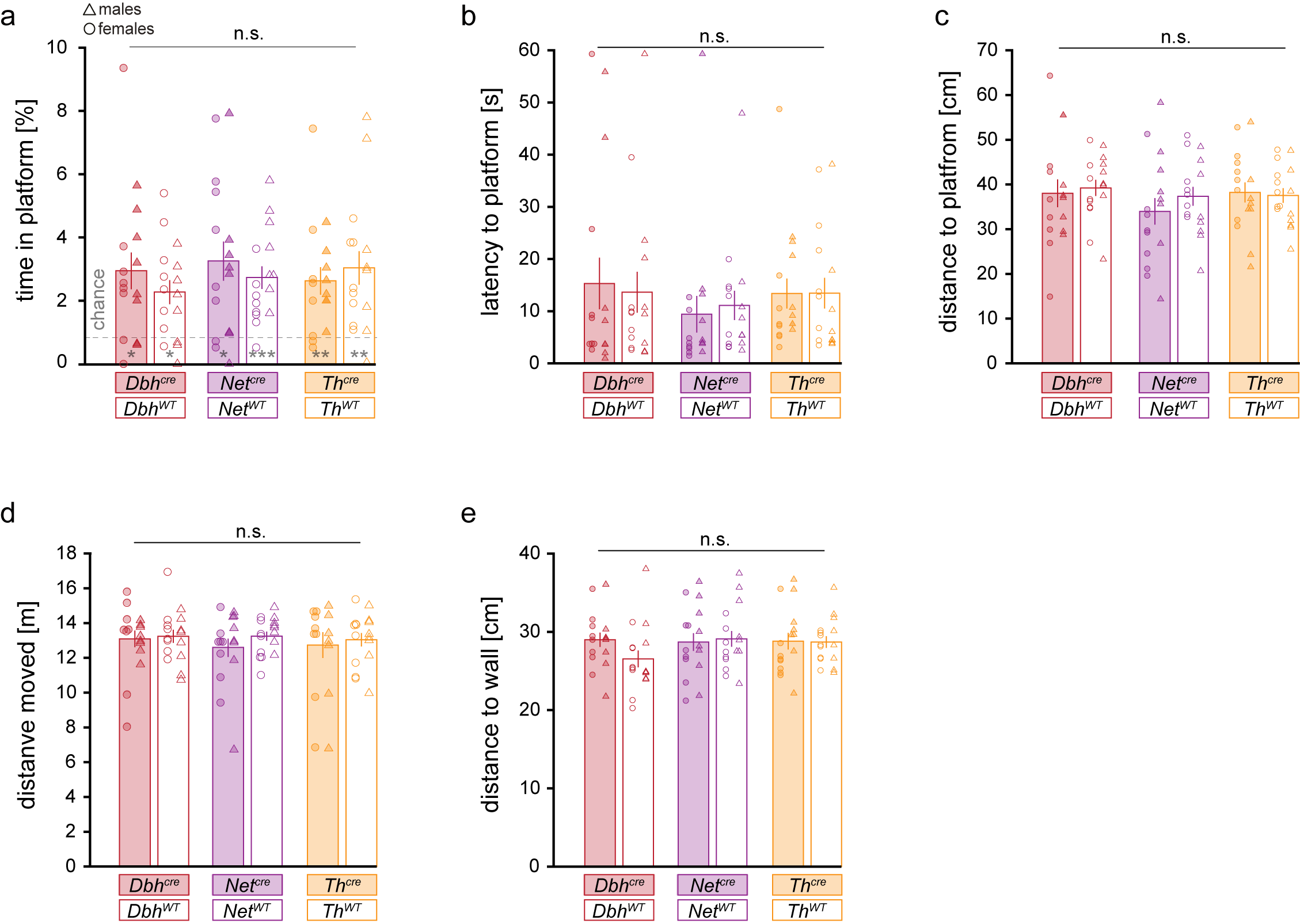

Supplement: S14 Fig — No effect of genotype could be detected on spatial memory as approximated by (a) the time spent in the platform area, (b) the time needed to reach the platform, and (c) the mean minimal distance to the platform. No effect of genotype could be detected on locomotion behavior as approximated by (d) the total distance traveled. No effect of genotype could be detected on (e) the average distance to the wall, suggesting comparable thigmotactic behavior of mice. All data is depicted as mean ± standard error of the mean. n.s. = not significant. n = 8 females (circles) and 8 males (triangles). */**/***, depicted in panel a, for p < 0.05/0.01/0.001, respectively, tested against chance level. Numerical data underlying this figure can be found in S1 Data. (TIF) [file pbio.3003228.s018.tif]
