# Supplementary material for: A comparison of viral strategies and model systems to target norepinephrine neurons in the locus coeruleus reveals high variability in transgene expression patterns
Source: PLoS Biol. 2025 Jul 7;23(7):e3003228. doi: 10.1371/journal.pbio.3003228 (PMC12233902; doi:10.1371/journal.pbio.3003228)
Supplement: Attachment — Submitted filename: Response_to_reviewers_Wissing_et_al.pdf [file pbio.3003228.s021.pdf]

Targeting Norepinephrine Neurons of the Locus Coeruleus: A Comparison of Model Systems and Strategies

– Response to Reviewers –

Reviewer #1: In this paper, the authors conducted a comparison of different methods to genetically target LC NE neurons. They compared three genetically modified mouse lines: DBH-Cre, NET-Cre, and TH-Cre, as well as cell targeting using a synthetic PRS×8 promoter. Firstly, they compared the transduction efficiency (comprising efficacy and specificity) of the different approaches and found that DBH-Cre, NET-Cre, and PRS×8 induced more efficient transduction than TH-Cre. More interestingly, in terms of ectopic expression, DBH-Cre showed the lowest level compared to all the other models, with TH-Cre showing substantial ectopic expression in the peri-LC areas such as in the parabrachial nucleus. In addition, the authors screened the different Cre mouse lines behaviorally to investigate differences in stress and anxiety (using open field and EPM assays) and in the formation of episodic and spatial memory (using Y-maze and Morris water maze assays). No genotype-related behavioral differences were found in any of the behavioral assays considered. Overall, the data are very well presented. The authors conducted essential controls to verify their findings, and they effectively quantified the success rates of LC targeting. I believe that this study would make a great contribution to the LC field, and some of their findings might explain reproducibility issues among different laboratories studying the LC using different targeting approaches. Thus, I am in favor of publication. However, I have some comments which I think could further strengthen the results.

We thank Reviewer #1 for the detailed summary of our findings and for the appreciation of our study and its impact on the field. We are confident that we can address their remaining concerns as outlined below, and that these suggestions will further strengthen our manuscript.

(1) The substantial ectopic expression, especially in the peri-LC area, in the case of the TH-Cre mouse line, is very worrisome for its use in studying the LC, as the authors clearly explain in their discussion. It is interesting, however, that areas such as the parabrachial nucleus appear as sites of ectopic expression in both the TH-Cre and PRSx8-mediated expression cases. Do you think this is just by chance?

We do not have a clear explanation for the ectopic expression in the parabrachial and the vestibular nuclei. We do not assume a molecular predisposition of these neurons to TH- or PRS×8-mediated transgene expression. Since parabrachial and the vestibular nucleus frequently expressed fluorophores upon injection of non cre-dependent constructs (e.g., hSyn-eGFP in Fig. 3e-j and Fig. S9, most pronounced upon injection of low volumes or serotype rAAV2/2, or hSyn-DO-tdTomato in Fig. 4c), these regions

might rather be simply better accessible for the viral suspension, likely due to the neuronal density in these regions and a high density in fiber tracts (mainly composed of densely packed axons). We have added a corresponding statement to our discussion (line 607-614; please note that all line references in this rebuttal letter refer to the manuscript version including tracked changes).

Moreover, as the authors cite in their discussion, areas around the LC have been shown to express TH (although these might be dopaminergic rather than noradrenergic), which could explain the ectopic expression in the TH-Cre mouse line. However, those areas do not show up in TH staining. Do you think that the GFP expression in those peri-LC areas is induced only by leaky expression of Cre due to the way the TH-Cre mouse line was generated? Could one imagine that the TH-staining procedure has some limited sensitivity to those cells, and they are indeed expressing TH endogenously?

As shown in Reviewer Figure 1 below, TH staining is not detectable even when saturating the red channels, in strongly GFP expressing cells. Vice versa, there are TH-positive cells, which have no detectable GFP-expression. This data suggests that even if there might be endogenous TH expression in these cells, it must occur at very low levels as compared to the TH^+ cells identified by our anti-TH staining. While we cannot prove the mechanism of ectopic expression with certainty, we believe that it is not simply explained by low sensitivity of our antibody staining, as eGFP and TH expression is clearly de-coupled (see also the next response).

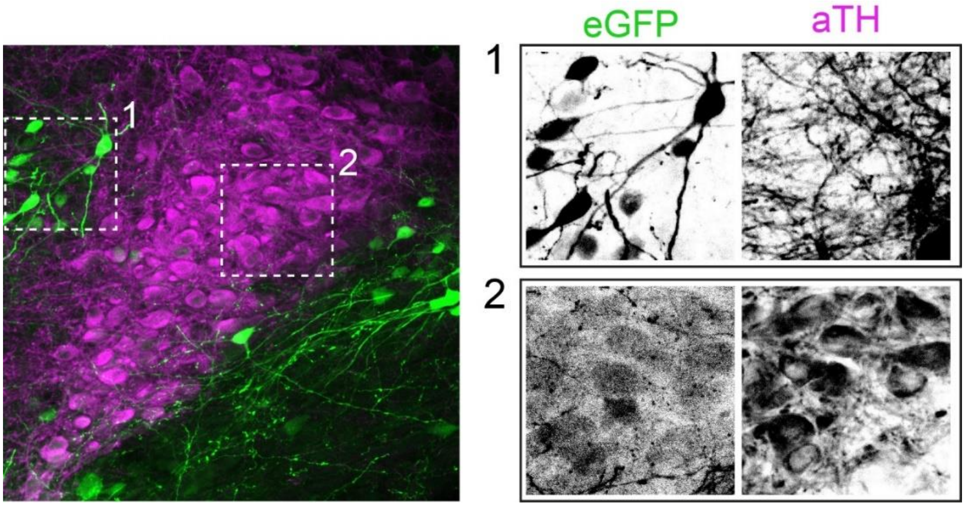

Reviewer Figure 1: Individual color channels with saturated signal. Left: Overlay image a *Th*^{cre} mouse injected with rAAV2/9-CAG-DIO-eGFP, stained against GFP (green) and TH (magenta). (1): Individual channels of exemplary neurons that ectopically express eGFP. No co-localized signal is detected by the aTH-staining, even when saturating the channel. (2): Individual channels of exemplary neurons that express TH (bottom right). No/very low co-localized signal is detected in the green channel. This becomes even more evident when comparing the fluorescence intensity of cells to the fluorescence intensity of background in the eGFP channel of examples 1 and 2.

In the native-GFP expression (Supplementary Figure 4), it seems that the presumably TH-negative cells medial to the right LC express eGFP more strongly than the LC cells. How do you explain this?

We have now investigated the relationship between GFP expression levels and native TH expression, as estimated from immunostainings against TH, in more detail. We indeed found that relative levels of unspecific eGFP-expression in non-TH cells (i.e., false positive neurons) are higher than eGFP expression levels in TH-positive cells in the Th^{cre} mouse line, while this is not the case in all other lines or wild-type animals (Dbh^{cre}, Net^{cre}, PRS×8). Here, GFP expression was higher in TH-positive cells compared to TH-negative cells. Although this does not directly answer the question why there is strong GFP expression in TH-negative neurons in the Th^{cre} line, these results clearly demonstrate that transgene expression upon viral transduction is decoupled from native TH expression in Th^{cre} mice, but not in the other lines or with the PRSx8 approach. We have included this analysis in our manuscript (Fig. 1f, Fig. S2) and added corresponding text to our results (line 174-189).

In addition, the number of eGFP-positive cells in the LC (Figure 1) in the case of the TH-Cre mouse line is surprisingly low. Why do you think this is the case, given that those cells are TH-positive? How do you explain this?

Indeed, the efficacy of LC transduction in the Th^{cre} mouse is surprisingly low in some animals. Generally, we have observed larger variability across animals in Th^{cre} mice as compared to the other approaches, as evident from the jitter in Figure 1d. While we do not have a mechanistic explanation to this, it unlikely results from genetic drift in some of our breeding animals, as breeding of our mice occurred with heterozygous Th^{cre} males and wildtype females, and we found mice with high and low efficacy even in the same litter. We have added a corresponding statement to our results section (line 133-136) and included additional examples of raw data from Th^{cre} (Fig. S1). These examples highlight a case of high efficacy and relatively high specificity versus a case in which low efficacy and low specificity, and corroborate our observations of less reliable transgene expression in Th^{cre} mice.

I think the authors address some of these concerns in their discussion, but given the strong message this conveys to the field—regarding one of the most used mouse lines for LC targeting—I think there must be no doubt about that specific result.

We fully agree and we are confident that the additional raw data provided in Fig. S1 and Reviewer Figure 1, as well as the additional analysis in Fig. 1f and Fig. S2 (which clearly demonstrates de-coupling of virus-mediated eGFP expression from native TH expression in Th^{cre} mice) strongly supports our conclusions. To further ensure that our experimental approach and our analysis pipelines lead to solid conclusions, we have

included an additional experiment, in which we have injected cre-dependent eGFP into the ventral tegmental area of *Th^cre^* mice. As previously reported by Lammel and colleagues[1], cre-dependent transduction of VTA neurons in the same mouse line (*Th^cre^*, Jax: 8601) results in substantial ectopic transgene expression, with an overall specificity of eGFP expression of 59% (Fig. 1H in Lammel et al). We could reproduce these experiments with our approach (average specificity of 60.7 ± 6.8 %, n = 25 slices from 4 mice), as shown in Fig. S6 and summarized in the results (line 210-214). Thus, our experimental pipeline results in comparable conclusions to published human observations and further strengthens our confidence in the conclusions derived from LC data in the current manuscript.

(2) In the case of the jRGECOa fiber photometry and the issue of isosbestic excitation which the authors explained, another way to compute the F0 would be to fit a 5th degree polynomial to the red signal and use this as an F0. I do not think this would change their observations; it is just a proposition.

We appreciate the reviewer's considerations on the isosbestic controls of red fluorescent calcium indicators, which we have given some thought ourselves, and experimented with different approaches to correct our signals (e.g., fitting of exponential, double-exponential, polynomials of different degree; high-pass-filtering, PCA-based corrections). While polynomials in principle work well, we are unsure which degree of a polynomial is ideal, as this might depend on the type of data (e.g., the presence of slow signal modulations which might be subtracted from the signal, as we observe in different experiments being done in our lab), and hence an adjustment based on (subjective) visual inspection is necessary. Since we are unsure about the rationale behind choosing a polynomial of 5th degree, we have decided to implement a double exponential fit as suggested in a review by Simpson et al. [2], which accounts for two different bleaching rates, one resulting from bleaching of the indicator, and one resulting from bleaching of the fiber autofluorescence. However, we have tried multiple options, and as the reviewer already suggested, none of them changes the observations in a qualitative way. Either way, we suggest that the best way to provide an isosbestic control – in addition to reporting ΔF/F – is to show the functional channel side by side with the control channel, as we have done in Fig. 6e.

Reviewer #2: Here, Wissing et al. present a comprehensive characterization of commonly used transgenic+viral strategies for targeting norepinephrine-expressing neurons in the rodent Locus Coeruleus (LC). Their justification for this work is two-fold: 1) LC is a brain region with diverse functionality and disease relevance but has been understudied due to the challenge of accurately targeting it in vivo. With advances in transgenic and recombinase-dependent AAVs, there is an increasing motivation to study LC function, and number of tools have been developed for researchers to use, but distinctions in their performance have not

been succinctly performed. 2) Recent characterization of other neuromodulatory transgenic lines have revealed behavioral abnormalities caused by Cre expression, an important caveat that could compromise functional characterization of targeted cell types. To address these issues, the authors perform a series of experiments that include quantification of Cre-dependent AAV expression in LC across transgenic lines, behavioral assessment of transgenic lines, and introduction and functional testing of PRSx8-dependent calcium reporters developed in their lab. Overall, I thought this study was well done, with rigorous experiments and data analysis, careful consideration and discussion of various results and caveats, and well-organized text and figures. It was very enjoyable to read!

Thank you.

A few specific strengths: I found the Cellpose-based analysis of LC very impressive! Due to the density of NE+ neurons in LC, it can be very challenging to accurately quantify cell numbers in this region, and thus, it is often not thoroughly done in published papers. I think the strategy presented here could be broadly useful to the field, especially as the authors also present validation/comparison with manually-analyzed images. I would encourage the authors to ensure that the methods are detailed enough such that others can replicate these pipelines for their own studies.

We appreciate the reviewer's comment on the usefulness of our analysis pipeline. We will upload both the customized *CellPose* model and the corresponding MATLAB-scripts to quantify transgene expression together with our paper on Github, so that the approach can be used by other laboratories as well. The use of our pipeline is explained in the Methods section of our manuscript and commented in our MATLAB scripts, while the use of *CellPose* is very well documented by the original authors (both in the original papers, and on their website https://www.cellpose.org/ and tutorials, https://www.youtube.com/watch?v=5qANHWoubZU). With the publication of documented code and the description in the Methods section, we are confident that other researchers will be able to replicate our pipeline.

The description of success rates regarding LC targeting was also thoroughly done. Again, I would suspect that this phenomenon has been observed by many in the field, but without such careful dissection of possible causes or suggested resolutions.

We agree. While working on additional experiments for the paper revision, we have increased the sample size for animals that were injected with more lateral coordinates (i.e., ± 1.1 instead of ± 0.9 mm relative to Bregma), leading to higher success rates of bilateral transgene expression. Motivated by this reviewer comment, and given that our manuscript is considered as a "Methods and Resources" article by the editorial team of *PLOS Biology*, we have expanded our original supplementary figure S6 (in the first submission) and moved it to the main manuscript (Fig. 4).

The authors summarize a wide range of studies succinctly, such that I anticipate this manuscript could serve as a useful resource for the field. For instance, their discussion of efficacies and titers of AAVs used across studies is very helpful. However, I would also suggest a few areas where the manuscript could be strengthened to improve the rigor and usefulness of these studies. 1) I think consideration of how serotypes and promoters affect LC infectivity needs to be addressed experimentally, rather than just discussed. I understand why it's not possible for the authors to extensively test all conditions that might influence efficacy and efficiency of LC infection, but even an initial comparison would greatly increase the usefulness of these experiments for the broader community:

We recognize the reviewers concern regarding additional parameters that can affect transgene expression patterns, such as viral serotypes or promoters, and have performed additional experiments to systematically evaluate their impact (as well as the impact of injection volume) as outlined below. We have summarized the results of these experiments in a new Figure 3, as well as in the results section of our manuscript (lines 258-337).

> a) Experimental comparison of the results they report here with AAV2/9 with another commonly used serotype …maybe AAV2/2 since it has been reported to have more restricted spread (PMID: 25240284)?
>
> Given the substantial ectopic expression observed in some approaches, we recognize the need to restrict the viral spread more closely to the LC. As the reviewer pointed out, this can be achieved by changing the viral serotype (e.g., rAAV2/2). To quantify the viral spread, we decided to inject an unconditional fluorophore (hSyn-eGFP) in wildtype mice to quantify viral spread independently of cre-dependent expression. This is mainly because conditional fluorophores could only be visualized if transduced brain regions are indeed ectopically expressing the transgene, and hence might underestimate the true viral spread. To investigate the effect of the viral serotype, we have compared injections of 300 nl of rAAV2/9-hSyn-eGFP (i.e., the serotype we have used in our study) to injections of 300 nl of rAAV2/2-hSyn-eGFP as suggested by the reviewer, and indeed found significantly reduced viral spread upon injection of rAAV2/2. In a different approach to reduce the viral spread, we investigated the effect of injection volume, and injected either 50, 100, or 300 nl of rAAV2/9 into the LC. Here, we found that injection of lower volumes significantly reduced viral spread. Together, these datasets suggest two options to restrict viral spread, which could be implemented to reduce ectopic expression in brain regions distant to the LC when relying on model systems that otherwise show ectopic expression (Fig. 3e-j).

b) Related, I think inclusion of an additional AAV reporter that utilizes a different promoter would greatly improve the study. I would suggest hSyn as many neural circuit-related AAV tools make use of it due to its small size. Also, I think it would be sufficient to compare performance of an hSyn-DIO-eGFP AAV to CAG-DIO-eGFP AAV in the AAV2/9 serotype only; no need to also expand this condition to multiple serotypes.

To verify whether our results also translate to the human synapsin promoter, as suggested by the reviewer, we have performed injections of rAAV2/9-hSyn-DIO-eGFP (i.e., a construct most comparable to the rAAV2/9-CAG-DIO-eGFP used in our other experiments) into the LC of Dbh^{cre} mice (n = 7) (Fig. 3a-d). We have decided to restrict our experiments to just this mouse line because (1) as opposed to Net^{cre}, it is freely available from Jackson Laboratories, (2) as opposed to Th^{cre} it results in high specificity and efficacy of transgene expression and (3) to keep animal numbers within a reasonable range. In these experiments, we found that expression patterns upon cre-mediated transgene expression are comparable between the CAG and hSyn promoter, both in terms of efficacy and in terms of specificity. This data suggests that also constructs based on the commonly used hSyn-promoter can be used to target the LC of Dbh^{cre} mice with high efficiency and that the less specific CAG promoter does not compromise specific expression.

2) I was confused by the PRSx8 results shown in Figure 1. In the image in 1c (bottom right corner), it looks like there's a lot of GFP+/TH- cells (so a lot of non-specific labeling), but this doesn't seem to be reflected in the metric reported in Figure 1d, where the samples seem to cluster much more closely to NETCre and DbhCre samples. I'm wondering if the authors expanded Figure 1 to include bar graphs of the specificity and efficacy values separately (or included this information as a supplementary figure) it would be easier to visualize specificity differences across samples and conditions?

Indeed, it is true that the Dbh^{cre} approach is significantly more specific than the PRS×8 approach (which is also indicated in panel 1d of our previous figure 1 by a single asterisk between Dbh^{cre} and PRSx8 on the x-axis), which was, however, not the case for Net^{cre}. To indicate these differences in efficacy and specificity more intuitively, we have removed the 2D-space plot in Figure 1d and instead visualize specificity and efficacy in bar graphs (Fig. 1d-e).

3) (minor). Some of these tools (ThCre and PRSx8) are also frequently utilized in rats, but no direct comparisons are made in the paper. I understand why it might be beyond the scope of the paper for the authors to experimentally test this (e.g., compare PRSx8-eGFP expression in mice vs. rats or DIO-eGFP

expression in ThCre mice vs ThCre rats). But at a minimum, I think there could be some discussion on whether they expect the tools to perform differently across model systems based on published studies, especially since they do such a good job compiling data across papers in other topics of the discussion.

We thank the reviewer for the appreciation of our comprehensive discussion. We now dedicate a section of the discussion specifically to rat models of LC-NE targeting (line 782-796), also including additional references.

Reviewer #3 (Jorge Miranda-Barrientos): In the manuscript "Targeting Norepinephrine Neurons of the Locus Coeruleus: A Comparison of Model Systems and Strategies," Wissing and collaborators compare various methods for selectively targeting Locus Coeruleus Norepinephrine (LC-NE) neurons. The authors examine three of the most commonly used Cre-driver lines (DBH-Cre, TH-Cre, and NET-Cre), along with a recently developed synthetic viral construct that utilizes a promoter derived from the human DBH promoter (PRSx8). To assess selectivity and specificity in the Cre-driver lines and wild-type animals injected with the PRSx8 viral vector, the authors injected a Cre-dependent virus encoding eGFP into the LC and asses its expression into the LC. The authors report variations in efficacy and specificity across the different targeting methods, noting similar performance among the DBH-Cre, and PRSx8-virus, while observing markedly lower efficiency and specificity in Cre-specific viral transduction within TH-Cre animals. Furthermore, the authors observed no behavioral abnormalities (anxiety, fear, and working memory) in any of the Cre driver lines. Finally, the authors developed and validated two viral vectors with the PRSx8 promoter to track LC NE neuronal activity using GCaMP8m (Green spectrum) and GRECO (red spectrum). The data presented by Wissing and collaborators shows that selective targeting of LC-NE neurons is achievable using DBH-Cre, and PRSx8 viral transduction, while cautioning experimenters to consider viral vector serotypes, tropisms, and the external validity of these data relative to specific experimental designs.

The information presented in the manuscript is highly relevant, as selectively targeting LC-NE neurons is crucial for understanding the cellular mechanisms underlying a wide variety of behaviors involving LC-NE. Furthermore, the lack of specificity in tools aimed at selectively targeting neuronal populations can introduce significant confounding factors and therefore such tools need to be validated and direct contrasted between the different options available. Overall, the manuscript is well written, and the data presented is convincing. The manuscript is easy to follow, with most of the experiments being logical and well-controlled. The supplementary data presented demonstrate the rigorous controls for most of the experiments. That being said, there are a few considerations that the authors might want to address before publication.

Considerations:

* The development of novel two viral vectors for assessing LC-NE neuronal activity using the PRSX8 platform is of great utility for the field. Although the pupillometry data gives support to the photometry data, it would be nice that the authors compare the data obtained with these novel viral vectors with a cre-dependent viral vector in one of the cre-driver lines that show similar specificity and efficacy to the PRSx8 viral vectors.

First, we thank the reviewer for pointing out the value of our study and the utility of our tools for the field. We acknowledge the concerns with respect to signal specificity of our PRS×8-based calcium indicators. To address them, we designed a new experiment, enabling direct comparison of PRS×8- and *Dbh*^{cre}-mediated expression of calcium indicators. To this end, we have injected PRS×8-jGCaMP8m into the LC of a *Dbh*^{cre} mouse, while injecting the spectrally compatible jRGECO1a in a cre-dependent manner in the contralateral LC (hSyn-DIO-jRGECO1a). We then performed dual-site photometry recordings from both LC while monitoring the animal's pupil, allowing to directly compare PRS×8- and *Dbh*^{cre}-mediated expression of calcium indicators on the functional level. The results of these experiments revealed strong correlations between both calcium indicators, and a similar temporal pattern of LC activity preceding pupil dilation. In addition, different control experiments excluded the possibility of motion and hemodynamic artefacts as well as spectral crosstalk (data shown in Fig. 6a-g, results described in lines 512-539). Hence, we are confident that PRS×8-jGCaMP8m-inferred calcium activity is reporting LC-NE activity with great specificity. As this dual-expression experiment was quite challenging to perform, and given that PRS×8-jRGECO1a has been used in a different study by our lab where it has been thoroughly validated[3], we decided to perform the analysis only for this combination of the indicators.

Since we decided to publish the PRS×8-jRGECO1a in the meantime in the study mentioned above, and encouraged by the reviewer's remark on the utility of the PRS×8 viral vectors for the field, we have decided to rather include an optogenetic actuator for specific LC activation. Thus, we have created a construct expressing the red-light sensitive channelrhodopsin ChrimsonR under control of the PRS×8 promoter (Fig. 6h-l, lines 540-548). PRS×8-ChrimsonR-tdTomato is spectrally compatible with PRS×8-GCaMP8m, and together these tools allow for all-optical manipulation and read-out of LC activity, independent of the genetic background of the mouse (or other animals).

* In that same line, since the efficacy and specificity of the viral vectors depend on the construct and the proteins encoded, it would be nice if the authors show data about the specificity and efficacy of their developed viral vectors.

We have now quantified the efficacy and specificity of these tools in the animals reported and stated it explicitly in the results section (lines 517-519 and 543, Fig. 6b, Fig. 6i). As the quantification of this data was harder to achieve in an automated way (fluorophore expression and the outline of cells were not as nicely delineated as compared to the expression of eGFP) we have added a corresponding note of caution to the methods section (lines 972-975).

* It would be informative if the authors included pictures showing the targeting of the optic fiber over the LC for the photometry recordings in figure 4.

We have now included microscopy images of whole coronal sections in which lesions from the fiber optics can be seen above the LC, as suggested by the reviewer (Fig. 6b, Fig. 6i). In one example, not the whole fiber tract is visible, so we chose the slice on which the part of the lesion terminating above the LC is most clearly visible (Fig. 6i).

* To aid clarity of results and data presentation, in Figure 1d the authors might want to consider switching the axis, so efficacy appears in the left X axis and specificity appears in the lower Y axis. This might help the readers to interpret the data presented as normally higher values are presented on the upper right-hand side of the graphs.

We recognize the reviewers concern regarding switching of the x-axis in Figure 1d. To support interpretation of the data, we have removed the 2D space from Fig. 1d and instead visualize efficacy and specificity are separately using bar graphs (Fig. 1d-e).

References:

1. Lammel, S. *et al.* Diversity of Transgenic Mouse Models for Selective Targeting of Midbrain Dopamine Neurons. *Neuron* **85**, 429–438 (2015).
2. Simpson, E. H. *et al.* Lights, fiber, action! A primer on in vivo fiber photometry. *Neuron* **112**, 718–739 (2024).
3. Maheu, M., Donner, T. H. & Wiegert, J. S. Serotonergic and noradrenergic interactions in pupil-linked arousal. Preprint at https://doi.org/10.1101/2025.03.26.644382 (2025).
